# Monthly Record of Deaths in the City of Buffalo

**Published:** 1854-08

**Authors:** Jas. Newman

**Affiliations:** Health Physician


ART. VII.—*Record of Mortality in the City of Buffalo, for the Month of June,* 1854. By J. M. NEWMAN, Health Physician.

| DISEASES. | No. | Males. | Females. |
|---|---|---|---|
| Abscessus Hepaticus, | 1 | | 1 |
| Accidens, | 3 | 3 | |
| Apoplexia, | 3 | 2 | 1 |
| Asphyxia Immersorum, | 7 | 7 | |
| Bronchitis, | 3 | 1 | 2 |
| Cancer Pulmonalis, | 1 | | 1 |
| Cholera Asphyxia, | 8 | 7 | 1 |
| " Infantum, | 1 | 1 | |
| Cynanche Trachealis, | 5 | 2 | 3 |
| " Tonsillaris, | 1 | 1 | |
| Debilitas, | 6 | 3 | 1 |
| Diarrhœa, | 3 | 2 | 1 |
| Dysenteria, | 6 | 2 | 4 |
| Eclampsia, | 12 | 5 | 5 |
| Enteritis, | 3 | 1 | 1 |
| Febris Puerperarum, | 2 | | 2 |
| " Scarlatina, | 4 | 2 | 2 |
| " Typhoid, | 1 | | 1 |
| " Typhus, | 3 | 1 | 2 |
| Hydrops Cerebri, | 9 | 2 | 7 |
| Hyperæmia Cerebri, | 2 | 2 | |
| " Pulmonalis, | 1 | | 1 |
| " ———? | 1 | | 1 |
| Hypertrophia et morbus Valvularum Cordis, | 1 | | 1 |
| Gangræna consecutus Impetigo, | 1 | 1 | |
| Intemperantia et Delir. Tremens, | 4 | 3 | 1 |
| Marasmus, | 1 | 1 | |
| Natus Mortuus, | 11 | | |
| Negligentia, | 2 | 1 | 1 |
| Pertussis, | 2 | 2 | |
| Phthisis Pulmonalis, | 18 | 9 | 7 |
| Pneumonia, | 2 | 1 | 1 |
| " Typhoid, | 2 | 2 | |
| Ramollissement Cerebelli, | 1 | 1 | |
| Rubeola, | 4 | 3 | 1 |
| Scrophula, | 1 | 1 | |
| Senectus, | 3 | 1 | 2 |
| Syphilis Hereditarius, | 1 | | 1 |
| Ignotus, | 23 | 9 | 12 |
| | 163 | | |

| | |
|---|---|
| Males, | 79 |
| Females, | 64 |
| Sex not given, | 20 |
| Total, | 163 |

### REGISTER OF MORTALITY—(Continued.)

### AGES.

| | | | |
|---|---|---|---|
| Still-born, | 11 | Between 6 " " 12 " | 11 |
| 1 day, | 2 | " 12 years and 20 years, | 10 |
| Between 1 day and 15 days, | 4 | " 20 " " 30 " | 12 |
| " 15 days and 30 days, | 1 | " 30 " " 40 " | 18 |
| " 1 month and 3 months, | 5 | " 40 " " 50 " | 7 |
| " 3 months and 6 months, | 5 | " 50 " " 60 " | 8 |
| " 6 " " 9 " | 6 | " 60 " " 70 " | 5 |
| " 9 " " 12 " | 6 | " 70 " " 80 " | 3 |
| " 1 year and 3 years, | 25 | Ages not given, | 6 |
| " 3 years and 6 years, | 18 | | |
| Total, | | | 163 |

### NATIVITY.

| | | | |
|---|---|---|---|
| American, | 45 | Hollander, | 1 |
| English, | 6 | Swiss, | 1 |
| Scotch, | 3 | Norwegian, | 1 |
| Irish, | 16 | Colored, | 5 |
| German, | 47 | Country not given, | 34 |
| French, | 4 | | |
| Total, | | | 163 |

### CORRECTION.

Several serious errors inadvertently crept into the printed report of the mortality for the month of May, given in the July number of this Journal.

Below we give this part of our tables corrected, and desire those who may feel interested in this branch of medical statistics, to make a note of the same.

| DISEASES. | No. | Males. | Females. |
|---|---|---|---|
| Paralysis, | 2 | 1 | |
| Phlebitis, | 1 | | 1 |
| Pleurisy, | 1 | | 1 |
| Phthisis Pulmonalis, | 30 | 14 | 16 |
| Phrenitis. | 1 | | 1 |
| Pneumonia, | 9 | 9 | |
| Rubeola, | 8 | 2 | 6 |

